# Metal loss defect detection and depth estimation using multi-spectral image analysis of cooling excited steel specimen with corrosion

**DOI:** 10.1038/s41598-025-88528-w

**Published:** 2025-07-04

**Authors:** Shamendra Egodawela, Amirali K. Gostar, H. A. D. Samith Buddika, W. A. N. I. Harischandra, A. J. Dhammika, Mojtaba Mahmoodian

**Affiliations:** 1 School of Engineering, RMIT University, Melbourne, 3001 Australia; 2 Faculty of Engineering, University of Peradeniya, Peradeniya, 20400 Sri Lanka

**Keywords:** Non-destructive testing, Corrosion severity estimation, Multi-spectral imaging, Machine learning, Engineering, Materials science, Physics

## Abstract

Imaging techniques have considerably improved corrosion-induced metal loss defect detection and severity estimation in recent decades. Even though the detection of defects using imaging techniques in steel is well established, determining the severity remains difficult due to the necessity of estimating the depth information of the defect from 2-dimensional image data. This study used a steel test specimen with artificial defects of varying depths and diameters, subjected to accelerated corrosion. A Multi-Spectral Imaging setup observed the specimen’s spectral response at different temperatures following a cooling excitation. Reflected intensities at specific wavelengths indicated defect presence and allowed quantification of corrosion-induced metal loss. Principal Component Analysis and machine learning regression were used to transform discrete defect depths into continuous assessments. Support Vector Regression, Decision Tree Regressor, Random Forest Regressor, Gradient Boosting Regressor, and a Feedforward Neural Network (FNN) were tested for this task. The FNN showed the best results in solving the regression problem with a least Root Mean Square Error of 0.2829 and an R^2^ score 0.976. The 700 nm–900 nm range was identified as the optimal wavelength span for spectral imaging.

## Introduction

Material loss due to corrosion causes local stress concentrations in steel members, reduction in the ultimate load-carrying capacity, and reduction in fatigue life of structures when subjected to external loads^1,2^. Techniques for quantifying metal loss defects due to corrosion can be broadly categorized into Direct Methods, which provide quantitative measurements of metal loss or corrosion rate, and Indirect Methods, which infer the presence of corrosion based on environmental or material changes^3^. Direct methods involve an inspector visually assessing the condition of corrosion to measure the depth of corrosion using micrometer needle-point gauges, ultrasonic or laser measurement devices^4^. Indirect methods provide qualitative insights into corrosive environments, enabling non-intrusive assessments over larger areas. Indirect methods comprise of Non Destructive Testing and Evaluation (NDT & E) methods such as Eddy current arrays^5^, Guided wave ultrasound devices^6^, Magnetic flux leakage testing^7^, Acoustic emission devices^8^ and Radiographic testing^9^. These are based on electrochemical principles that require multiple sensor placements on the surface under inspection, reducing their feasibility for on-field applications^10^. Imaging as an Indirect method for corrosion detection alone, can be a trivial task due to the distinct hue, shape and texture of corrosion products on the surface. Detection of corrosion on collected image data can be done by image processing techniques such as edge detection^11^, color space analysis^12^, numerical analysis^13^, texture analysis on gray level features^14^, object detection and segmentation using convolutional neural networks^15^.

Imaging methods for inspecting large areas rapidly, without the necessity of direct contact or immersion, make them lucrative alternatives to Direct methods. Techniques such as Visible light imaging (400–700 nm) Infrared Thermography (IRT) at IR wavelengths (700–1000 nm), Terahertz imaging at Terahertz wavelengths (3 mm and 300 $\upmu$m) employ a specific band of the EM spectrum for imaging. However, determining the severity of metal loss defects using imaging remains a difficult task due to the necessity of inferring depth information from 2-dimensional image data^5,6,16,17^. For this, IRT remains the most successful imaging technique to quantify metal loss at present^10,17^ through the spatio-temporal evolution of surface temperature providing insights into both surface and sub-surface defects. Using an external excitation source, thermal IR cameras capture thermograms that track the spatio-temporal evolution of surface temperature as the material transitions from excitation to ambient conditions. Unlike Visible light imaging, which is limited to surface-level defects, and Terahertz imaging, which struggles with metallic materials due to high reflectivity, IRT effectively detects anomalies by leveraging heat conduction properties, making it highly suitable for metallic defect characterization. Heating excitation methods commonly used in active IRT include optical thermography with xenon or laser flashes^18^, vibrothermography using mechanical perturbations^19^, and induction thermography with pulsed eddy currents^20^. Eddy current thermography combines eddy current testing is used for conductive materials by visualizing heat anomalies caused by induced eddy currents^20^. It enables real-time defect detection but is limited by skin depth and surface variations. Herein, cooling excitation offers several key advantages over traditional heating methods. It provides uniform thermal gradients, avoiding localized hotspots that obscure sub-surface details, and minimizes thermal reflections, enhancing imaging accuracy^21^. Cooling can also be a more cost-effective solution in high-temperature industrial environments, leveraging existing thermal conditions to reduce energy and equipment needs^3^. Additionally, it avoids thermal stress or damage to metallic materials, making it suitable for sensitive components, and enables realistic NDT & E without altering progression dynamics^22^. Despite the success of IRT, temperature measurements are affected by ambient and surface conditions, including direct sunlight, reflected energy from the background, emissivity, roughness of material and inherent temperature of the material, leading to erroneous thermograms^17^. More importantly, IRT exclusively works in IR range with IR range cameras, which may lead to overlooking obvious defects that are even detectable in visible light^10^.

**Fig. 1:**
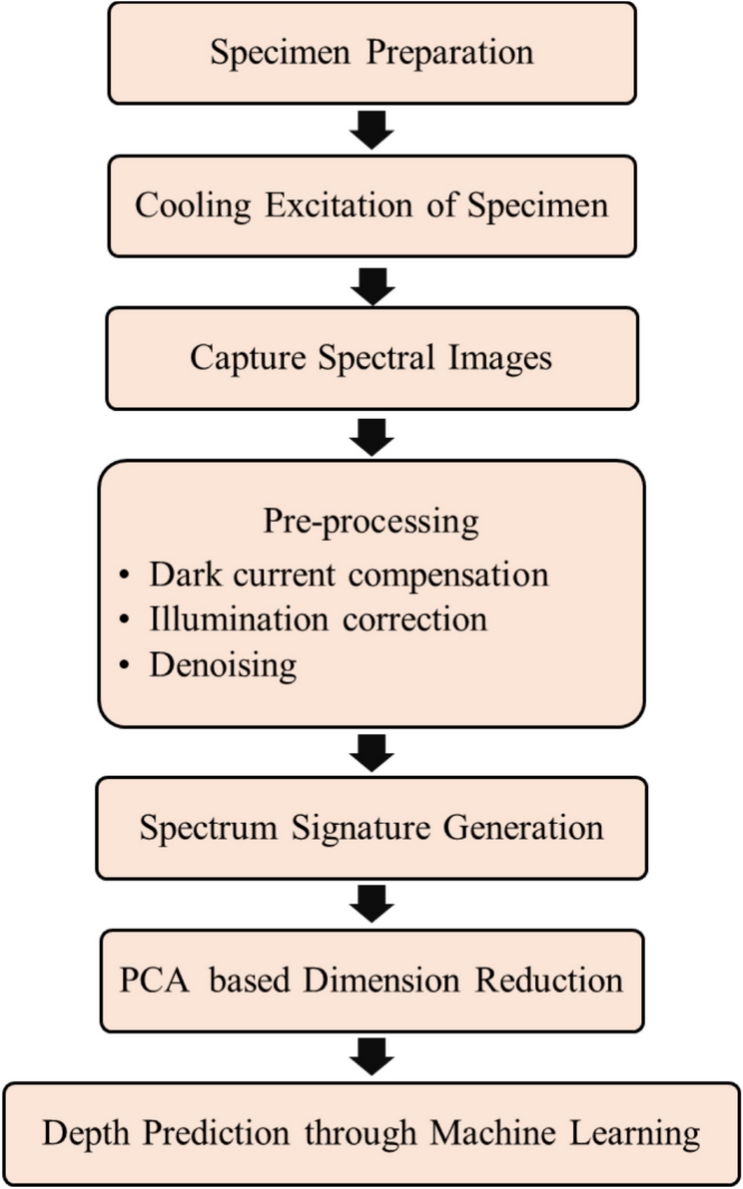
A block diagram illustrating the proposed methodology for detection and severity estimation of corrosion-induced metal loss defects.

Multi-Spectral Imaging (MSI) addresses these problems by imaging at multiple bands and fusing the information together to better represent the material under inspection. Due to the ability of MSI to capture the complete spectral response of the sample, it has been extensively used in food quality assessment^23^, biomedical industry^24^, art preservation and counterfeit detection^25^ and many other NDT & E applications^26^. MSI applied for corrosion evaluation is limited to detection of corrosion, via lowering visual ambiguity^27^ and finding the exact chemical root of corrosion by differentiating based on the chemical composition of corrosion products^28^. However, work remains to be done on using MSI for metal loss severity estimation using the depth of the defect. This is due to the lack of an external excitation (similar to active IRT) in existing MSI setups to enable depth extraction.

In this work, the authors perform MSI with a cooling excitation to extract depth information of corrosion defects for metal loss severity estimation. As proof of the concept, a steel specimen with artificially created flat-bottom defects was cooled to sub-ambient temperatures, and multi-spectral images under reflectance mode were collected. It was found that the reflected intensities at certain wavelengths can indicate not only defect presence but also provide clear means of quantifying metal loss in terms of the depth of the defect. In addition to the imaging system results, the paper presents a probabilistic method to quantify corrosion-induced metal loss using spectral data. Spectral data were collected for different temperature values after the thermal excitation at multiple wavelengths. The high dimensional spectral data were subjected to a dimension reduction process using Principal Component Analysis (PCA)^29^ followed by a machine learning regression process using Support Vector Regression (SVR) Decision Tree Regression (DTR), Random Forest Regression (RFR), Gradient Boosting regression (GBR) and Feedforward Neural Network (FNN) were used to quantify the defect depths. The main contributions of this paper include:

- Development of a Multi-Spectral Imaging System: The study introduces a novel MSI setup for detecting and quantifying metal loss defects in corroded steel specimens. This setup uses cooling excitation to extract depth information, critical for severity estimation.
- Innovative Methodology for Depth Estimation: The proposed method involves cooling the steel specimen to sub-ambient temperatures and capturing multi-spectral images at various wavelengths during the warming phase. This approach helps identify defects based on their spectral response at different temperatures.
- Machine Learning Application: The paper employs machine learning techniques, specifically PCA for dimension reduction and regression models including SVR, DTR, RFR, GBR, FNN to predict the depth of corrosion defects from multi-spectral data.
- Optimal Wavelength Identification: The study investigates the spectral responses of corrosion defects across multiple wavelength ranges to determine the most effective span for detecting and analyzing defects using MSI.
- Validation of Corrosion Detection and Depth Estimation: The proposed methodology is designed to evaluate metal specimens with varying defect depths and diameters, establishing its potential for NDT & E.
- Statistical and Theoretical Analysis: The paper provides a detailed theoretical explanation of the observed differences in reflected intensities between defected and non-defected areas based on energy interactions and temperature effects on reflectivity.

These contributions highlight the effectiveness and innovation of the proposed MSI system and methodology in improving the detection and quantification of corrosion-induced metal loss defects in steel structures.

## Experimental setup

The proposed methodology involves several steps as shown in Fig. 1. First, steel specimens with artificial defects of varying depths and diameters are prepared to simulate corrosion-induced metal loss. The specimen is then cooled to sub-ambient temperatures to enhance contrast between defected and non-defected areas. Multi-spectral images are captured across various wavelengths, collecting comprehensive spectral dataset. These images undergo pre-processing, including dark current compensation, illumination correction, and denoising to improve quality. Processed images yield spectral signatures that reveal defect locations. PCA applied for the generated spectral signatures reduces the complexity of spectral data, simplifying the analysis. Machine learning regression models are then used to predict defect depths from the reduced data, providing precise evaluations of defect severity. This method combines advanced imaging and data analysis to detect and quantify corrosion-induced defects in steel specimens effectively. Comprehensive details of the methodology are provided in the following section.

### Specimen preparation

**Table 1:** Chemical composition of ASTM A36 steel^30^.

Element	w/w (%)
Carbon (C)	0.25
Copper (Cu)	0.20
Silicon (Si)	0.28
Manganese (Mn)	1.02
Phosphorous (P)	0.04
Sulfur (S)	0.05
Iron (Fe)	Remainder

**Fig. 2:**
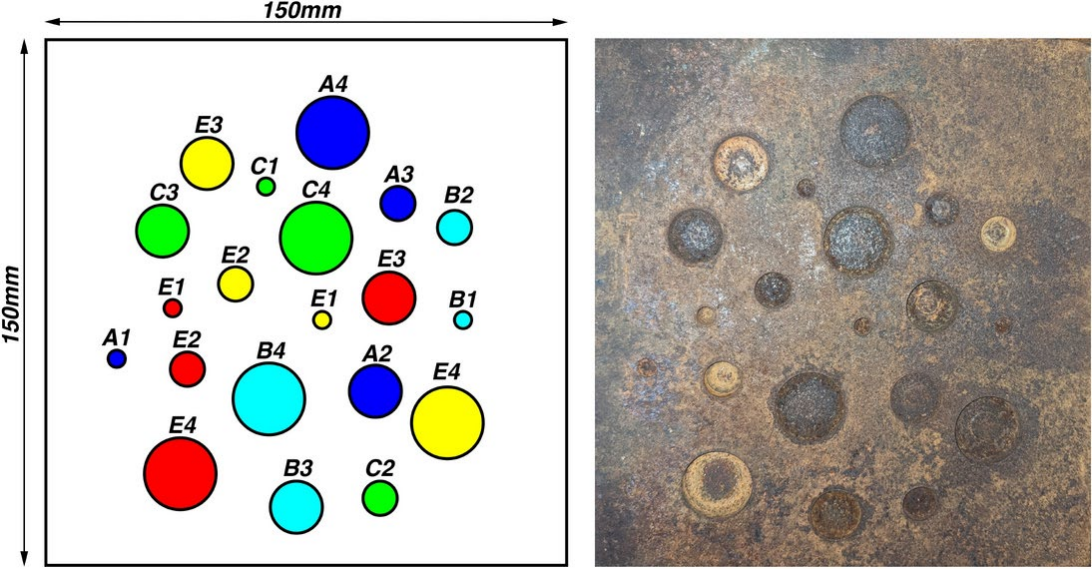
ASTM A36 test specimen dimension details with metal loss defects replicated as flat bottom holes with different diameters and depths.

American Society for Testing and Materials (ASTM) standard, A36 Carbon steel test piece of dimensions 150 mm $\times$ 150 mm $\times$ 10 mm was sourced to perform validation of the proposed method. ASTM A36 is the most commonly used structural steel for construction^30^. Table 1 shows the elemental chemical composition of A36 steel used in this experiment. As suggested in literature^3,31,32^, metal loss defects are replicated by machining randomly distributed, flat bottom, circular blind holes (impenetrable holes) with zero taper. These holes are commonly used in experimental setups because they provide controlled and reproducible geometries for validating defect characterization techniques. A twist drill with a multi-point drill-bit equal to the desired hole diameter was used to axially pierce the top surface of the work piece to a near-finished depth. Next, a counter-bore tool of the same diameter was used to obtain a flat and flush finish on the bottom surface. Figure 2 and Table 2 show the distribution of defects at different depths and diameters on the test piece. A mild degreaser with distilled water and acetone was applied using a dry cloth to remove the lubrication oil applied during the machining process. The contaminant-free test piece was subjected to an accelerated corrosion process using analytical grade concentrated HCl and bi-distilled deionized water. A 1M HCl solution was found sufficient to replicate surface corrosion on the machined surface overnight. A clear distinction was made in the specimens’ physical appearance before and after the corrosion process.

**Table 2:** Details of artificial defects on the steel test specimen.

	A				B				C				D				E			
Subgroup	1	2	3	4	1	2	3	4	1	2	3	4	1	2	3	4	1	2	3	4
Diameter (mm)	5	10	15	20	5	10	15	20	5	10	15	20	5	10	15	20	5	10	15	20
Depth (mm)	1	1	1	1	2	2	2	2	3	3	3	3	4	4	4	4	5	5	5	5

**Fig. 3:**
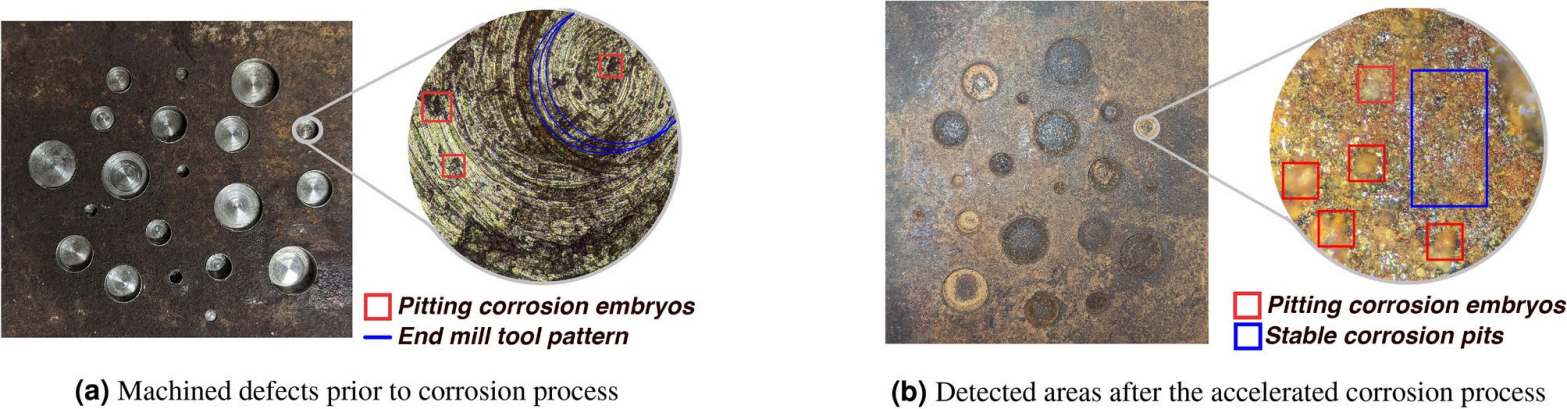
Metallographic Analysis of accelerated corrosion process shows clear evidence of corrosion. Following the accelerated corrosion process, an abundance of pitting embryos and stable corrosion pits were observed on the metal surface.

A metallographic examination was conducted using an optical microscope (ZEISS Axiom Lab.A1$\phantom{0}^{\hbox {TM}}$) to verify the corrosion process further. Corrosion pits were examined under zoom levels of 50$\times$ and 100$\times$, and evidence of corrosion pit embryos and corrosion nuclei were found on a uniform bed of passive oxide layer. Figure 3a shows the machined test piece before being subjected to accelerated corrosion, and Fig. 3b shows the test piece after the accelerated corrosion process.

### Cooling excitation setup

For the cooling excitation, the defective specimen was slow cooled to a temperature of 0 $\phantom{0}^{\circ }$C using a refrigerator (Fisherbrand$\phantom{0}^{\hbox {TM}}$ Isotemp$\phantom{0}^{\hbox {TM}}$ General Purpose Laboratory Refrigerator). The specimen was then placed inside the imaging system enclosure mentioned in the following section to reheat to ambient temperature of 24 $\phantom{0}^{\circ }$C. The surface temperature was measured in real-time using a thermocouple (Furukawa Electric Co., Ltd. Thermocouple JA75A4) in contact with the specimen surface. Images were captured from 0 $\phantom{0}^{\circ }$C to 24 $\phantom{0}^{\circ }$C in 2 $\phantom{0}^{\circ }$C increments. The temperature range of 0–24 $\phantom{0}^{\circ }$C and sampling at 2 $\phantom{0}^{\circ }$C intervals was chosen to reflect realistic ambient conditions, ensuring the method’s applicability in practical scenarios while capturing the spatio-temporal evolution of thermal gradients for accurate defect characterization.

### Imaging system

**Fig. 4:**
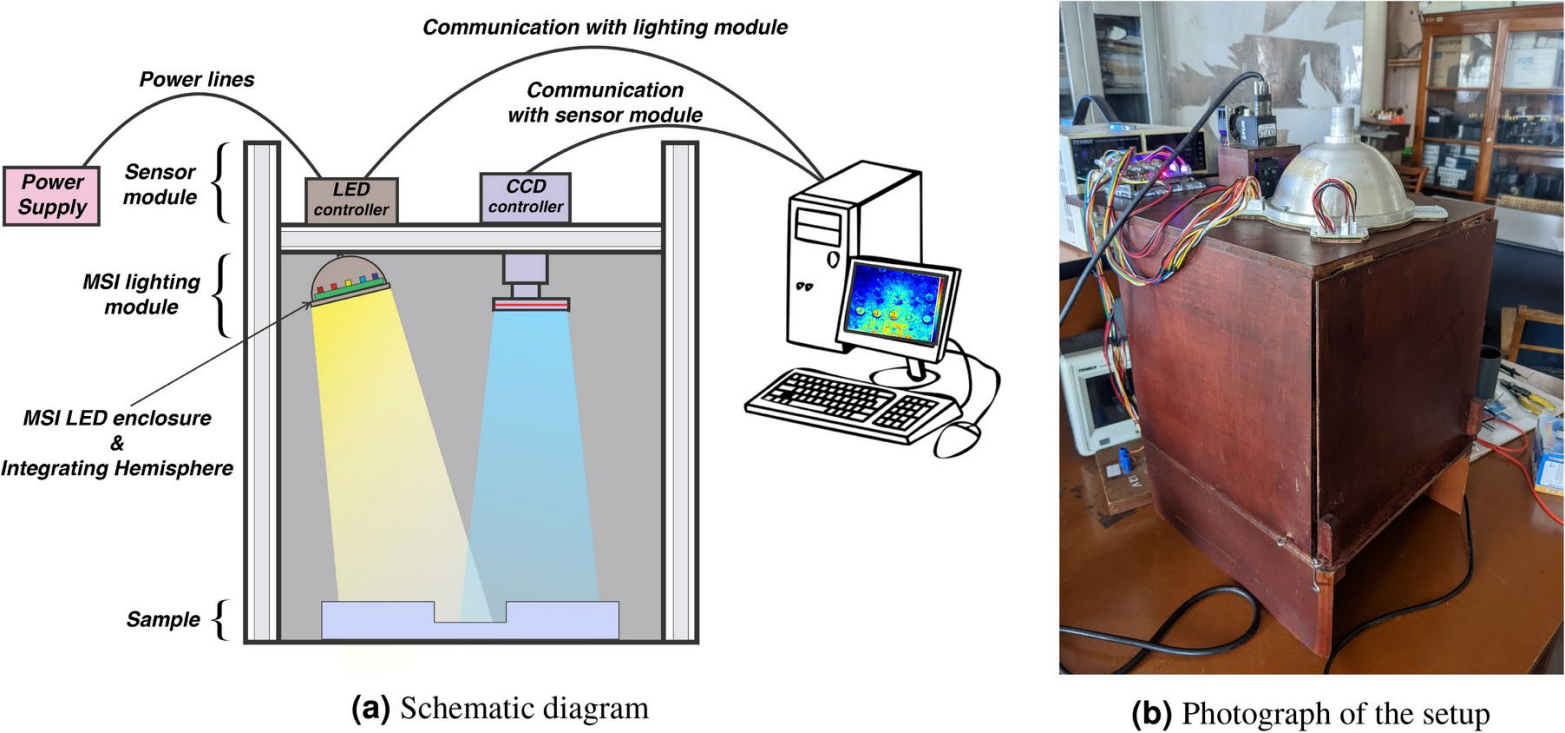
Multi-Spectral Imaging setup. (**a**) Schematic diagram illustrating the MSI system components and arrangement. (**b**) Photograph of the actual MSI system setup used in the study.

The works inspired the MSI system used in this study in food quality assessment research^33–35^ and Fig. 4 shows the complete imaging system. The monochrome imager employed in this study was a FLIR Blackfly S USB 3.1 by FLIR Systems Inc $\circledR$. Specifications of the camera are given in Table 3. The imaging system was interfaced with an edge computing processor via a proprietary frame-capturing software SpinView$\circledR$.

**Table 3:** FLIR Blackfly S monochrome camera details^36^.

Description	Value
Resolution	1280$\times$1024
Dimensions	[*W*$\times$*H*$\times$*L*] 29 mm $\times$ 29 mm $\times$ 30 mm
Frame Rate	60FPS
Sensor	Semi P1300, CMOS, 1/2”
Power Consumption	$\le$3W
Port	USB 3

**Fig. 5:**
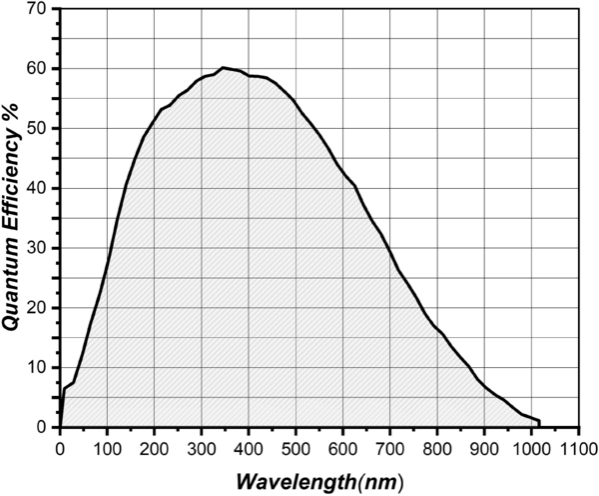
Monochrome camera Charge Coupled Device (CCD) response curve shows non-linearity across the wavelengths^36^.

The scene was illuminated using a patch of LEDs with varying wavelengths 365 nm, 405 nm, 473 nm, 530 nm, 577 nm, 621 nm, 660 nm, 735 nm, 770 nm, 830 nm, 850 nm, 890 nm, 940 nm. The range of LEDs chosen to cover the electromagnetic spectrum from UV to IR range as suggested by^26^ and according to the commercial availability. To compensate for the effect of non-uniformity in the sensor Charge Coupled Device (CCD) response curve shown in Fig. 5, power supplied to the LEDs was regulated using Pulse Width Modulation (PWM) regulator units. By lighting the LEDs brighter at ranges with a lower quantum efficiency of the CCD and dimmer at high-efficiency regions, a uniform power output from the CCD device across the wavelength was obtained. The LED lighting modulation circuit was powered by a stabilized 12V, DC, 50W power supply and 5V square wave switching was used with varying duty cycle to adjust the brightness of the LEDs. An integration sphere, as suggested by the work^37^ made of Aluminum, was used to provide spatially uniform illumination onto the scene.

Integrating spheres adds incident light from multiple light sources by evenly diffusing reflection on the sphere’s inner surface. When multiple LEDs of the same wavelength are lit up, an integrating sphere must add the light rays together and provide a more uniform illumination onto the scene^37^. The complete imaging system schematic shown in Fig. 4a, was placed inside a wooden chamber shown in Fig. 4b to prevent external light and reflections from influencing the scene lighting.

## Data processing

The data captured using the imaging system holds raw spatial and spectral information. However, the image data must be processed to extract useful information for defect detection and quantification. This section details the processing steps used for defect detection and depth estimation.

**Fig. 6:**
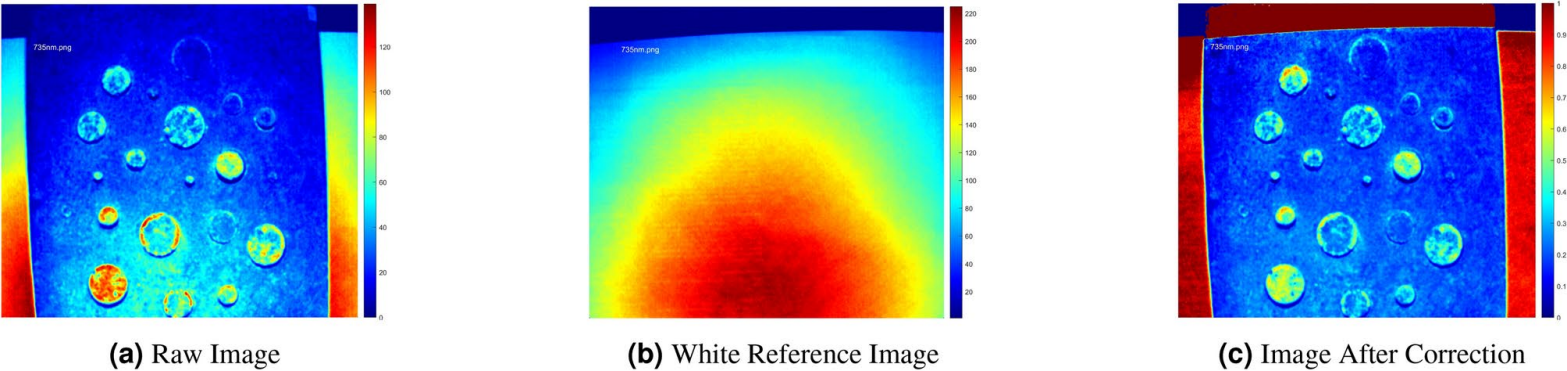
Correction of the spatial variations of the light source using the (**a**) Raw Image from the spectral imaging system and the (**b**) White Reference Image as suggested by^34^ and (**c**) the image after illumination correction.

### Image pre-processing

#### Dark current compensation

The presence of noise highly influences raw spectral image data. Dark current, also called dark signal or black level, is the signal generated by the CCD of the multi-spectral camera under zero illumination conditions^38^. This phenomenon is observed due to current leakages, imperfect charge transport, or electrons inherently generated and captured by the potential generating wells of the CCD due to thermal excitation^38^. To account for the fact, the dark current image was captured separately prior to capturing the image of interest and can be offset using the linear combination as mentioned in^34^ given by Eq. (1),

1 $$\begin{aligned} I_{xy}(\lambda )\ =\ I_{raw}(\lambda )-I_{dark}(\lambda ) \end{aligned}$$

where $I_{xy}(\lambda )$ is the dark current compensated image of wavelength $\lambda , I_{raw}(\lambda )$ is the raw monochrome image, and $I_{dark}(\lambda )$ is dark current image captured at zero illumination.

#### Correction of the spatial variations of the light source

LEDs of selected wavelengths were used as the lighting source of the imaging system. LEDs offer a high degree of directionality, giving rise to spatial illumination variation within the camera’s field of view. Such variation must be accounted for algorithmically to avoid actual defected areas localized in dimly lit areas from being overlooked. Equation (2) suggested by^34^ shows the ratio correction for dark current and illumination. Reference white reflection under uniform illumination for each wavelength was captured using a reference white reflection standard object placed on the camera’s focal plane. Care was taken to capture the white reference images slightly out of focus to ensure the same illumination characteristics as recommended by^39^. Figure 6 shows how the white reference images can be used to compensate for the spatial variation of the light on the scene using Eq. (2).

2 $$\begin{aligned} \rho _{xy}\left( \lambda \right) =\rho _{ref}\frac{I_{raw}\left( \lambda \right) -I_{dark}\left( \lambda \right) }{I_{white}\left( \lambda \right) -I_{dark}\left( \lambda \right) } \end{aligned}$$

where $\rho _{xy}(\lambda )$ is the illumination corrected monochrome image, $\rho _{ref}$ is the certified reflectance of the white reference object, $I_{raw}(\lambda )$ is the raw monochrome image, $I_{dark}(\lambda )$ is the dark current image captured separately, and $I_{white}(\lambda )$ is the white reference image. With $\rho _{ref}$ set to unity, it was observed that Eq. (2) implicitly resolves other illumination issues such as, differing gain values within the CCD matrix, a.k.a. CCD gain offset, mechanical and Optical vignetting caused by mechanical elements attached to the lens and the entrance pupil of the lens being obstructed from the lens barrel.

#### Contrast limited adaptive histogram equalization (CLAHE)

Illumination correction mentioned in the previous section performs appreciably at low Signal to Noise Ratio (SNR) settings. However, when high SNR is present, the ratio-derived image tends to have discontinuities as some pixel values may tend to be undefined values. A local operator limiting extreme peaks within the image was deemed necessary to mitigate the fact.

CLAHE is a derivation of Adaptive Histogra Equalization (AHE)^40^, wherein a local neighborhood is transformed using a transformation function to have a near-uniform histogram. The goal is to produce an image with a homogeneous contrast distribution within the operational window. However, in obtaining a near-uniform histogram, the algorithm amplifies noise by mapping low intensity values lower and high-intensity values even higher. As a result, when noise is present in the image, the neighboring pixel’s intensities may vary drastically. Therefore, a contrast-clipping limit is introduced to truncate the histogram prior to the mapping fuction, removing peaks that correspond to noise.

CLAHE algorithm with a 32 $\times$ 32 grid size was applied to the image to reduce illumination differences within the images by improving contrast in local areas. An empirical value for a clipping limit of 4 units was selected as a compromise between inherent peaks on the image due to defects against peaks occurring due to noise.

#### Median filtering for noise removal

As mentioned previously, the success of MSI techniques relies heavily on raw spectral data and the ensuing processing techniques. Here, raw spectral data revealed a heavy presence of speckle and salt-and-pepper noise was noticed. Speckle noise arises from the thermal interference on the imaging sensor during image acquisition. In contrast, salt and pepper noise is impulse noise generally caused by analogue to digital conversions and/or during data transmission. When speckle and salt-pepper noise are present, noise suppression using Non-linear 2D Median filtering has been extensively used in literature^41,42^ and was shown to be effective for this application. Equation (3 shows the Median filtering processing for a 2D image processing problem.

3 $$\begin{aligned} I^*[i,\ j] = \text {median} \left\{ I\left[ (i-k):(i+k),\ (j-l):(j+l)\right] \right\} \end{aligned}$$

where $I^*[i,\ j]$ is the denoised image, *k* and *l* are the spatial resolutions of the Median filtering window and *I* is the illumination corrected image prior to denoising.

### Dataset

**Fig. 7:**
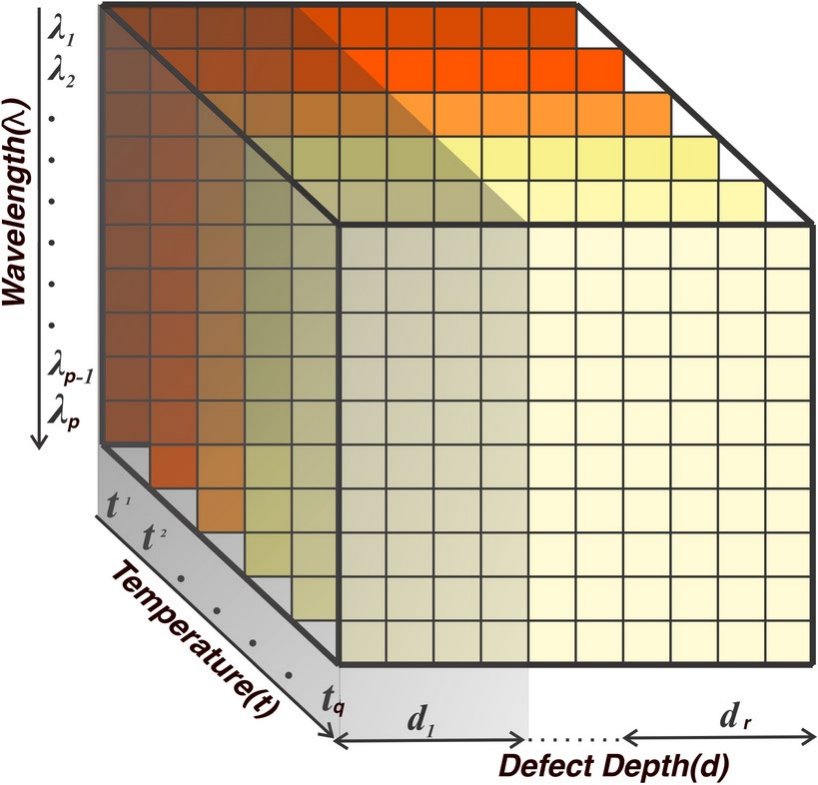
Data cube (*D*) of size $p \times q \times r$, consisting of spectral data placed according to the wavelength ($\lambda$), defect depth (*d*), and temperature (*t*) of each defect.

The calibrated spectral reflectance data of the imaging system after pre-processing were recorded, and a master dataset cube, henceforth referred to as *D* was created. Defected areas were hand-picked as pixel patches and global average pooling was used to generate a singular value representing each defect’s reflectance intensity. The representative values of reflectance for the defected areas were placed on *D*, according to the wavelength ($\lambda$), defect depth (*d*) and temperature(*t*) of the images captured. On the dataset *D*, the spectral dimension consisted of the wavelengths under observation as rows ($\lambda _{1}, \lambda _{2}$,..., $\lambda _{p-1}$, $\lambda _{p}$), defect depths as columns ($d_{1}, d_{2},\ldots , d_{r-1}, d_{r}$)and temperature the images were captured ($t_{1}, t_{2},\ldots , t_{q-1}, t_{q}$) at as the remaining dimension of the data cube. Figure 7 shows the layout of *D* in a graphical sense. The total size of the data cube was recorded as $p \times q \times r$.

In this study, a total of 2880 data points were collected with *p* = 12 wavelength ranges, *q* = 12 temperature values ranging from 0–24 $\phantom{0}^{\circ }$C in 2 $\phantom{0}^{\circ }$C intervals, *r* = 20 defected areas on the specimen. The training, testing and validation datasets to train and deploy the machine learning classifier suggested in the following section were generated by partitioning the master dataset *D* according to the ratio of 70:15:15. This ratio was chosen based on widely accepted practices in machine learning^43,44^. The training portion was utilized to train the regression models, while the validation portion was used for tuning the hyperparameters of each regression model. The test data was reserved exclusively for evaluating the performance of the models on unseen data, ensuring an unbiased assessment of their generalization capabilities. Here the training data points were chosen randomly from *D* to ensure the classifier had equal exposure to all defect depths.

### Multi-spectral data analysis

#### Spectrum signatures

Spectral signature is the variation of surface reflectance magnitude against changing spectral wavelength. The spectral signature indicates light interaction with the specimen under observation and can be representative of its optical and physical properties^34^.

To observe the change of reflectance due to the cooling excitation, a study of the spectrum signature of the defective steel specimen is conducted in this work. For a given temperature (*t*) in *D*, the plot of the elements in a row (*d*) against $\lambda$ gives the spectral signature. The process can be repeated for temperatures ($t_{1}, t_{2}, \ldots , t_{r-1}, t_{r}$) resulting in a clear picture of how surface reflectance magnitude changes with wavelength and temperature. The spectrum signature generation process results are shown in the Results section showed a noticeable difference in reflectance intensity, especially at the IR and Near IR wavelengths. However, overlaps in spectral bands limited separability, reducing regression performance. Therefore, spectral signatures alone did not classify each depth level satisfactorily.  Consequently, a PCA step was used to reduce the high-dimensional spectral data to a lower-dimensional latent space for machine learning regression.

#### PCA for dimension reduction of spectral data

PCA is a popular dimensionality reduction method in spectral analysis^45–47^ to capture the latent variables within a dataset. PCA functions by an underlying Eigen analysis to construct multiple orthonormal basis vectors that allow the identification of high-variance basis under the assumption that the higher variance would allow maximum class separability. Furthermore, PCA reduces data redundancy by minimizing cross-correlation between data and improving the discrimination between different classes^47^. The results of the PCA are detailed in Results section. Choosing the number of Principal Components (PCs) is a key step in PCA. The approach suggested by^48^ using a Scree plot was used to identify the number of PC directions required to capture the bulk of the information within the dataset. A Scree plot is created by plotting each eigenvector’s eigenvalue (i.e., explained variance) in descending order. The cumulative sum of these eigenvalues indicates the proportion of total variance captured. Typically, a threshold of 90% cumulative variability is deemed sufficient to capture information within the whole dataset^46^, and the number of PCs needed to reach the threshold was chosen for the regression analysis.

#### Machine learning regression to predict defect depth

The high-dimensional data points were reduced to a latent space using PCA, where the number of components was selected based on a cumulative variance threshold. This selection process, guided by a scree plot analysis, ensured the preservation of the majority of the feature information while reducing dimensionality. Machine learning regression analysis was then applied on the reduced dimension space to transform discrete defect depths into a continuous scale, enabling a more precise assessment of defect severity. SVR^49^, DTR^50^, RFR^51^, GBR^51^ and a FNN^52^ was tested for solving the regression problem. The performance of the regression models was assessed using three key metrics: Root Mean Square Error (RMSE) (4), Coefficient of Determination (R$\phantom{0}^{2}$) (5), and Explained Variance Score (EVS) (6). Together, these metrics offered a comprehensive evaluation of the models’ predictive capabilities. Details of the hyperparameters and the results for each model are provided in the Results section.

4 $$\begin{aligned} \text {RMSE} = \sqrt{\frac{1}{n} \sum _{i=1}^n (\hat{y}_i - y_i)^2} \end{aligned}$$

5 $$\begin{aligned} R^2 = 1 - \frac{\sum _{i=1}^n (y_i - \hat{y}_i)^2}{\sum _{i=1}^n (y_i - \bar{y})^2} \end{aligned}$$

6 $$\begin{aligned} \text {EVS} = 1 - \frac{\sigma ^2(y - \hat{y})}{\sigma ^2 (y)} \end{aligned}$$

where Target depth values : $y_i$, Predicted depth values : $\hat{y}_i$, Total number of data points : $n$, Mean of the target depth values : $\bar{y} = \frac{1}{n} \sum _{i=1}^n y_i$, Variance of target depth values : $\sigma ^2(y) = \frac{1}{n} \sum _{i=1}^n (y_i - \bar{y})^2$, Variance of residuals : $\sigma ^2(y - \hat{y}) = \frac{1}{n} \sum _{i=1}^n \left( (y_i - \hat{y}_i) - \overline{(y - \hat{y})} \right) ^2$, Mean of residuals : $\overline{(y - \hat{y})} = \frac{1}{n} \sum _{i=1}^n (y_i - \hat{y}_i)$.

## Theoretical reasoning of reflected intensity differences in defected areas due to cooling excitation

**Fig. 8:**
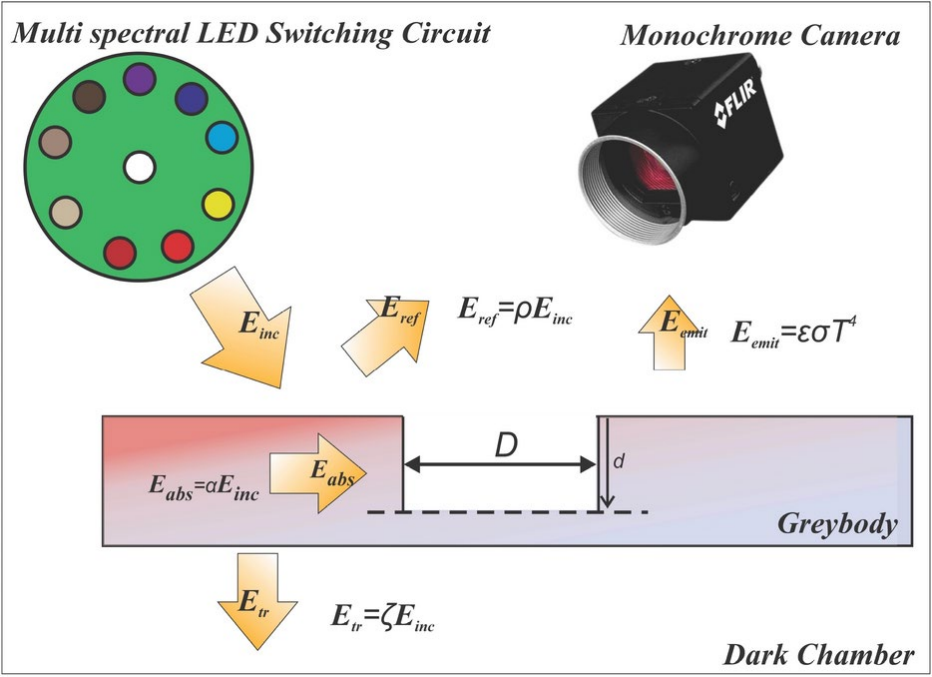
Energy interactions between the multi-spectral system and the sample under inspection. Here, the total energy captured by the image sensor is ($E_{sensor}$), The Emitted energy ($E_{emit}$) and the Reflected energy ($E_{ref}$) of the multi-spectral illumination.

In this section, clear reasoning is provided for the differences in reflected intensities in defected and non-defected areas appearing on MSI data. As shown in the coming sections, the reflected intensity differences give rise to defected areas being highlighted and visible on MSI data. This is done by considering the energy interactions between the steel specimen and the MSI source and the effect of cooling excitation on the said energy interactions. Figure 8 shows the energy interactions between the multi-spectral illumination and the specimen. As shown in the Figure, the total energy captured by the image sensor ($E_{sensor}$) given in Eq. (7) contains the Emitted energy ($E_{emit}$) and the Reflected energy ($E_{ref}$) of the multi-spectral illumination.

7 $$\begin{aligned} E_{sensor}=E_{emit}+E_{ref} \end{aligned}$$

The emitted energy ($E_{emit}$) is defined by the Stefan Boltzmann law^53^ given in Eq. (8). It is independent of the multi-spectral lighting or imaging wavelength but rather depends on the absolute temperature (*T*) and the specimen’s emissivity ($\varepsilon$). The reflected energy ($E_{ref}$) depends on the spectral reflectivity ($\rho$) at the given temperature (*T*) and the surface area of the reflected surface (*A*) and is given in Eq. (9).

8 $$\begin{aligned} E_{emit}&= \varepsilon \sigma AT^4 \end{aligned}$$

9 $$\begin{aligned} E_{ref}&= \rho \left( T\right) E_{inc} A\left( D,d\right) \end{aligned}$$

It can be rationalized that at zero illumination conditions, the captured image (a.k.a. the dark current image) contains only the Emitted energy from the specimen. However, since the dark current is removed during pre-processing using Eq. (1), it can be assumed that the emitted energy captured by the sensor is completely removed from the image data. Therefore, the images after the pre-processing steps contain only the reflected energy and none of the emitted energy from the specimen. In summary, the energy captured by the sensor is given according to Eq. (10) with $E_{emit}$ = 0 in Eq. (7) and $E_{ref}$ in Eq. (9).

10 $$\begin{aligned} E_{sensor}=\rho \left( T\right) .E_{inc}.A\left( D,d\right) \end{aligned}$$

Here, for a given incident light source, the reflected energy depends on two factors: the spectral reflectivity and the geometry of the reflected surface. The effects of cooling excitation and sample defects and its effect on $\rho (t)$ is explained in a heat transfer sense in Sections “Temperature differences in defected and non-defected areas due to metal loss defects” and “Temperature dependence of reflectivity”. Section Surface geometry discusses the effect of defect diameter and depth on *A*(*D*, *d*). Thereby explaining the differences between the intensity differences seen by the sensor $E_{sensor}$, in defected and non-defected areas.

### Temperature differences in defected and non-defected areas due to metal loss defects

Fourier’s transient heat conduction law^54^ given in Eq. (11) governs the spatio-temporal expectation of temperature for sound materials. A multitude of research work builds on this concept for different materials, geometries, and boundary conditions^54–56^. Deviations from ideal temperature expectations are considered an anomaly in the material, indicating the presence of defects.

11 $$\begin{aligned} \frac{\partial ^2T}{\partial x^2}+\frac{\partial ^2T}{\partial y^2}+\frac{\partial ^2T}{\partial z^2}+\frac{\dot{q}}{k}=\frac{\rho C_p}{k}\frac{\partial T}{\partial t} \end{aligned}$$

As suggested previously^54–56^, thermal diffusion mechanisms of heating and cooling are similar and follow the classical model as in Eq. (11). A defected specimen cooled to a steady temperature left in the ambient environment absorbs heat via incident radiation, and the temperature rises steadily until thermal equilibrium is established. This transient behavior has been studied thoroughly in literature as long pulse thermography, where specimens are subjected to a step energy source^57–59^. The 1-dimensional simplification for Equation 8 with no internal heat generation gives the spatial and temporal temperature variation in Eq. (12)^57^. The infinite series solution for the temperature expectation for a non-defected sample is given in Eq. (13).

12 $$\begin{aligned} \frac{\partial ^2T}{\partial x^2}=\frac{k}{\rho C_p}\frac{\partial T}{\partial t} \end{aligned}$$

where *T*is the temperature, *x* is the vector direction along the material where the temperature is measured, *k* is the thermal conductivity of the material, $\rho$ is the material density, $C_p$ is the specific heat capacity of the solid per unit volume, and *t* is the time. Here, $\alpha = \rho$ $C_{p}$, is known as thermal diffusivity^57^. For an infinitely long plate with a total thickness of 2*L*, $T_{i}$ is the initial temperature of the plate, $T_{1}$ is the step change of temperature at the walls of the plate, the solution of Eq. (9) by separation of variables is given by Eq. (13) with boundary conditions of *x* ranging from 0 to 2*L* and $t>$ 0 with $n = 1, 3, 5,...$^60^,

13 $$\begin{aligned} T\left( x,t\right) =T_1+\left( T_i-T_1\right) \frac{4}{\pi }\sum _{n=1}^{\infty }\frac{1}{n}\exp {\left[ -\left( \frac{n\pi }{2L}\right) ^2\alpha t\right] }\cdot \sin {\left( \frac{n\pi x}{2L}\right) } \end{aligned}$$

It has been shown that when metal loss defects are present, the diffusion rate ($\alpha$) of the energy pulse is non-uniform inside the material, causing a temperature gradient at defected locations^58,59^. As a result, temperature differences exist in defected and non-defected areas at a given time. Section Temperature dependence of reflectivity discusses the effect of differing temperatures in defected and non-defected areas affects $\rho (t)$ in Eq. (10), giving rise to defected areas being highlighted as shown in the coming sections.

### Temperature dependence of reflectivity

The temperature dependence of reflectivity $\rho (t)$ for various metals and alloys (for both polished and unpolished surfaces) has been discussed thoroughly in literature^61,62^. The modeling of light-metal interaction was pioneered by the works of Drude and Lorentz^63^. Herein, the Drude theory for free electrons is the most accepted complete theory, particularly for visible and IR ranges. Lorentz postulated that the optical properties of a metal depend on the plasma frequency of the metal, the optical frequency, and the relaxation time of electrons in the metal. The plasma frequency is affected in an inverse sense to electron-phonon

**Fig. 9:**
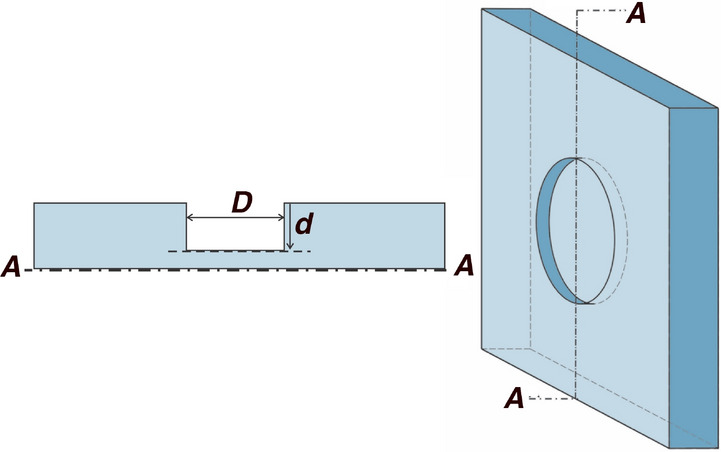
Artificial flat bottom defects generated on the steel specimen with the diameter (*D*) and depth (*d*) of the defects.

collision frequency, and the phonon collision population determines the temperature of the metal^63^. Therein lies the connection between the optical properties of metals with temperature.

However, an explicit and quantitative definition of reflectance against temperature is out of the scope of this work as it is affected by many physical factors such as type of material, surface finish, working temperature, pressure, etc. Therefore, for the scope of this study, the authors intend to assume that there is some variation in surface reflectance with temperature. This assumption gives reason for differing reflectivity values at defected and non-defected areas at varying temperatures, as explained in the previous section. Therefore, the defected areas are highlighted from the non-defected areas, as seen in the coming sections.

### Surface geometry

As per Eq. (9), the total reflected energy from the top surface of the specimen depends on the total surface area of the reflection surface. Figure 9 shows the geometry of the defected areas. The flat bottom defects can be modeled as an open cylinder, where Eq. (14) gives the total surface area of the metal loss pit. For a given temperature,

14 $$\begin{aligned} A\left( D,d\right) =\pi D\left( \frac{D}{4}+d\right) \end{aligned}$$

where *A* is the surface area of the defect, *D* is the diameter of the metal loss defect opening, *d* is the defect depth. Equation 14 shows that the surface area (*A*) increases linearly with the depth of the defect (*d*), and quadratically with diameter (*D*). Therefore, as per Eq. (9) for a given temperature, the total reflected energy increases linearly with *d* and quadratically with *D*. This gives rise to the fact that higher defect depth and diameter areas show higher intensity and look brighter on MSI data. This fact is evident from the MSI imaging results shown in the coming sections.

## Results and discussion

**Fig. 10:**
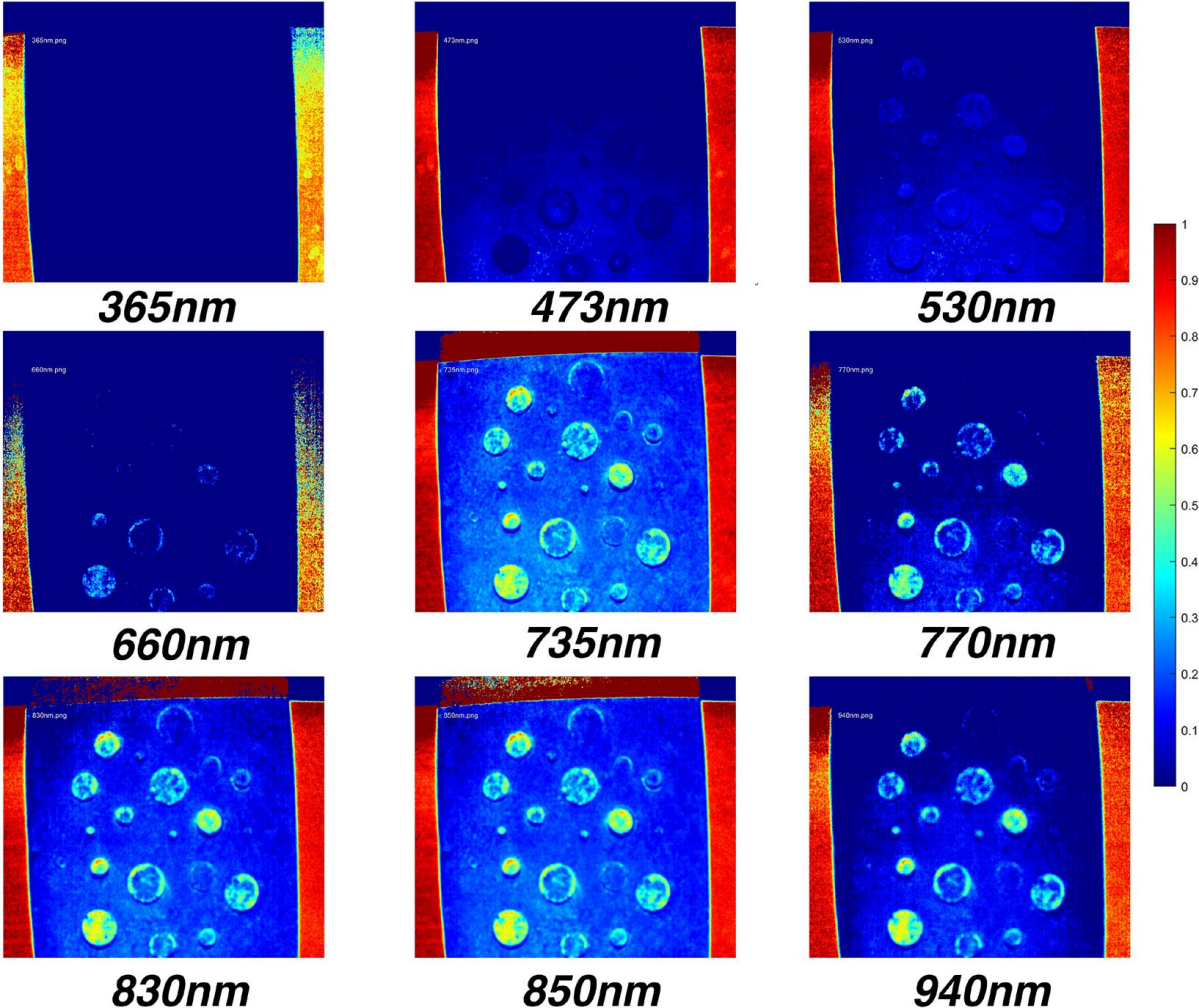
Multi-spectral image data of the defected steel specimen captured at 4 $\phantom{0}^{\circ }$C by the imaging system. The images show clear highlighting of defected areas at 700–900 nm range where no clear discernment can be made at the visible light range of 400–700 nm.

In this section, the results of the imaging system will be presented with the choice of 4 $\phantom{0}^{\circ }$C as the temperature value. Figure 10 shows the spectral image data at 4 $\phantom{0}^{\circ }$C after the pre-processing steps mentioned previously. The images show how defects are highlighted in the 700–900 nm range. Whereas the visible light range of 400–700 nm does not provide sufficient information to detect defects. The spectral images were used to construct the master spectral dataset *D*, and was used to perform the subsequent data analysis steps mentioned in previous sections.

**Fig. 11:**
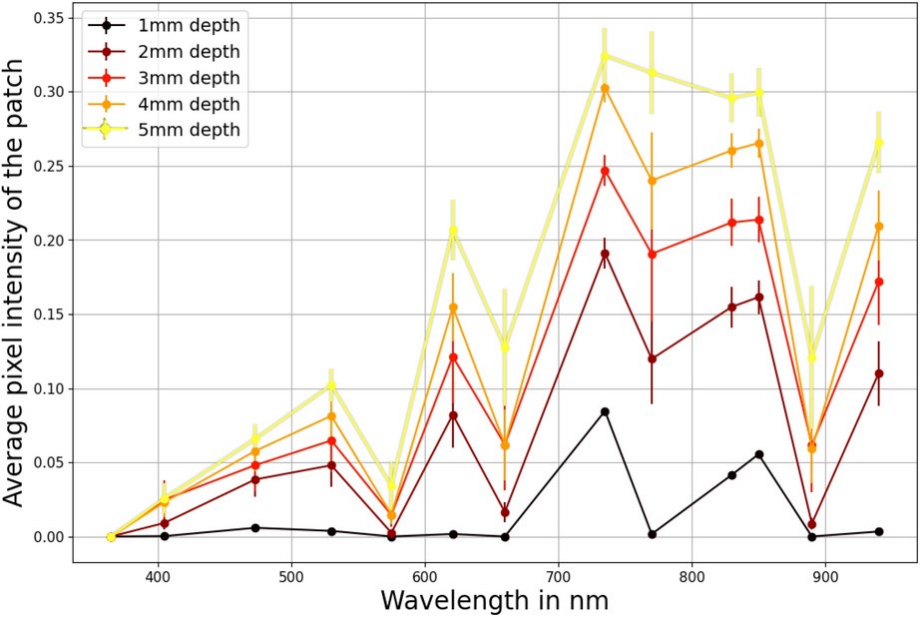
Spectrum signature of the specimen at 4 $\phantom{0}^{\circ }$C shows clear separability between spectral bands corresponding to different defect depths.

### Spectrum signatures

Spectrum signatures were generated to study the reflectance magnitude variation of defected areas. Figure 11 shows the spectral signatures at 4 $\phantom{0}^{\circ }$C temperature and the standard deviation of error at each depth. A key takeaway in this result is the fact that variation in the IR range (>700 nm) differs vastly from the visible range (400–700 nm). This may be due to reflectivity change effects in the IR range as suggested by Drude and Lorentz^63^. Metals typically exhibit poor reflectivity in the UV range due to their electronic structure which limits the interaction between incident UV light and the material’s surface^64^. This fact is evident where below 400 nm spectral images contained negligible or no meaningful information. 700–900 nm range was identified as a key wavelength span where depth estimation via the regression process could be done optimally. 735 nm wavelength was identified to give the most distinctive variations in the reflectance magnitudes at each depth value. This is evident from Fig. 11 where spectral bands at 735 nm wavelength are distinctly further apart.

Distinct differences in reflected intensities were observed across specific wavelengths at all observed temperatures. This phenomenon can be attributed to the fact that, although spectral reflectivity varies with temperature, the relative differences between defected and non-defected areas remain consistent. Additionally, other properties influencing reflected intensities, such as defect shape, remain constant throughout the temperature range. However, intermittent overlap between spectral bands at specific wavelengths highlighted the need for further refinement in depth estimation. To address this, PCA-based dimensionality reduction was applied, followed by machine learning regression techniques. Data from the UV range was excluded to concentrate on the remaining wavelengths, which offered more informative spectral responses. The detailed results of this analysis are presented in the following sections.

### PCA analysis

**Fig. 12:**
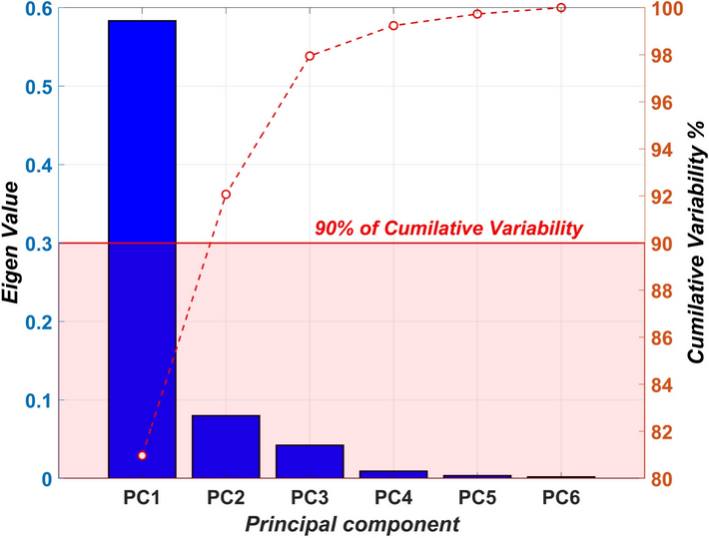
Scree plot of the PCA analysis with 90% accumulative variability contained within the first two PCs.

Choosing the number of principal components is integral to capturing the majority of a dataset’s latent variables. Figure 12 shows the Scree plot of the work, where the cumulative variability variation against the number of PCs is shown. It was concluded that choosing the first ($PC_{1}$) and second principal component ($PC_{2}$) directions were sufficient to capture 90% of the latent information within the dataset.

**Fig. 13:**
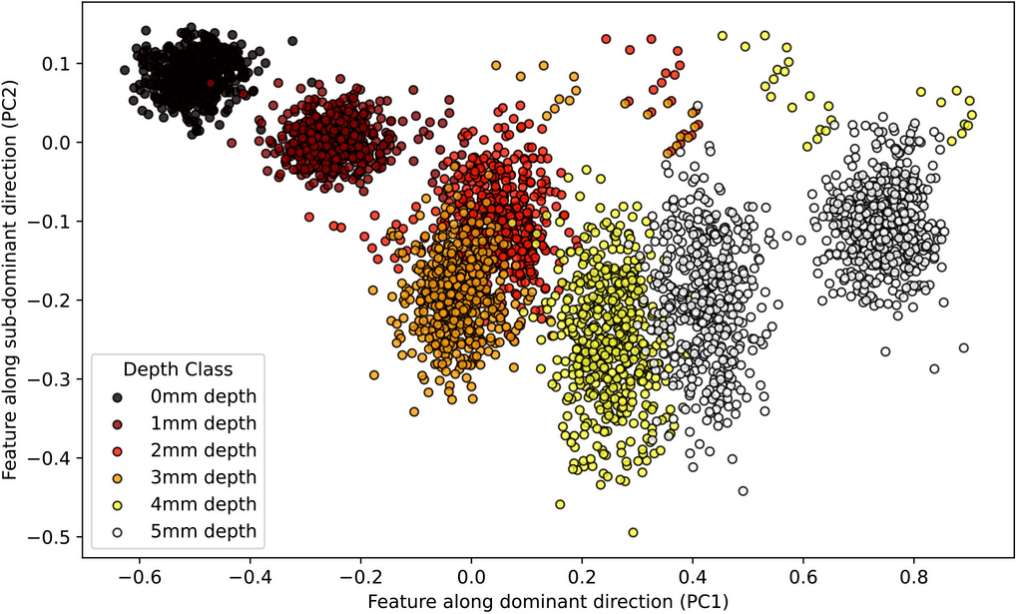
Training data points in *D* corresponding to 4 $\phantom{0}^{\circ }$C, mapped to the $PC_{1}$–$PC_{2}$ latent space after the PCA. PCA plot shows the demarcation of non-defected areas against the defected areas, leading to the machine learning regression process of the defect depths.

**Fig. 14:**
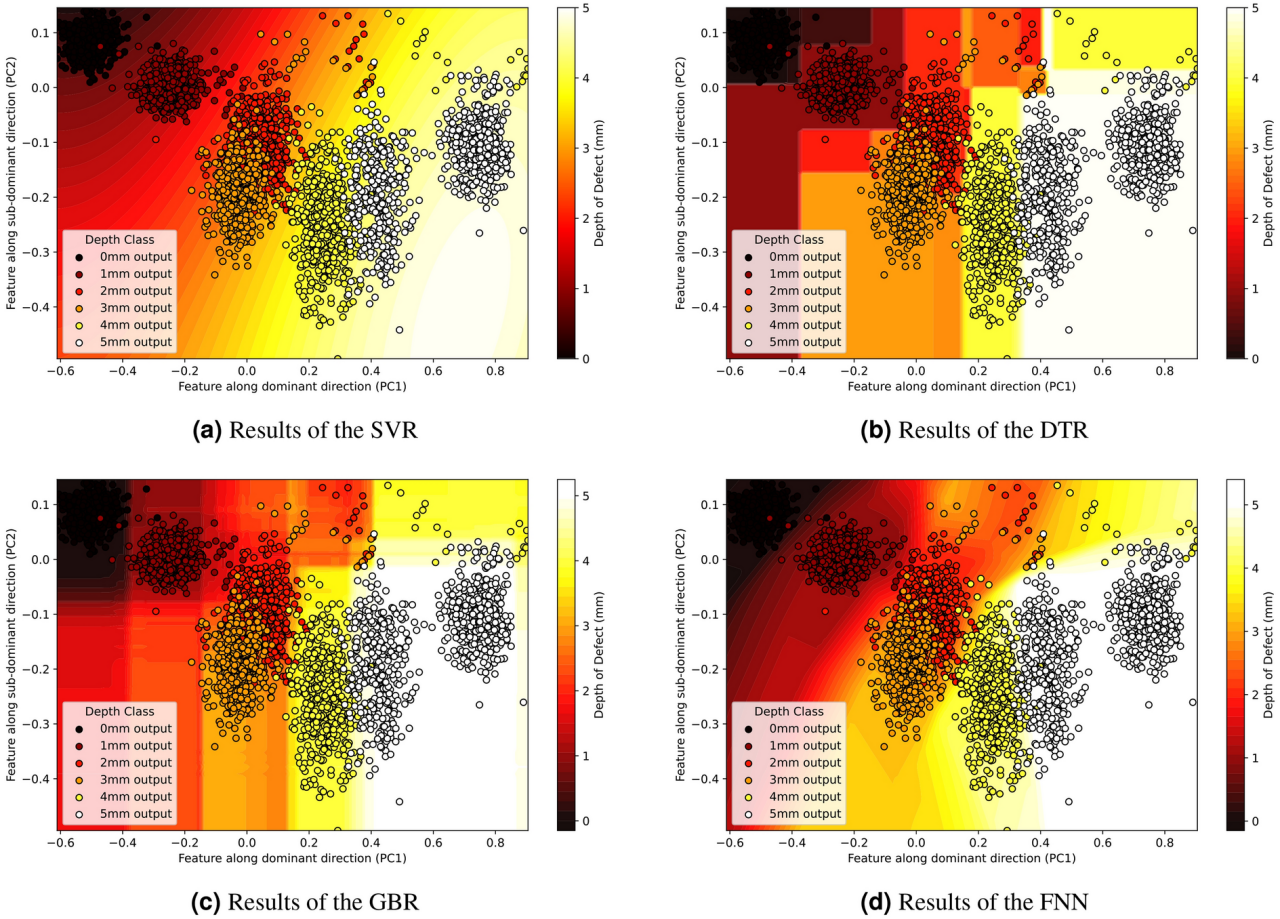
Results of the machine learning regression process for the tested methods.

Following the choice of the first two principal directions, existing 13-dimensional data space on the spectrum signature was mapped to the reduced latent two-dimensional space $PC_{1}$- $PC_{2}$. Figure 13 shows the apparent divergence of data points within the training portion of D. Clear separability in data shown in Figure 14 indicates that the imaging system successfully determined the depth of defects. It is important to note that the demarcation of the non-defected areas is unique in the $PC_{1}$- $PC_{2}$ space. However, there are some overlapping clusters in particular of 2 mm and 3 mm depths due to the visual ambiguity of the areas in the reflectance images. Machine learning regression techniques are suggested in the following section to estimate the depth data points in the $PC_{1}$- $PC_{2}$ latent space for continuous depth estimation.

### Machine learning models for depth estimation

The high-dimensional data points were mapped to a latent space using PCA. Then multiple machine-learning regression techniques were applied to transform discrete defect depths into a quantifiable and continuous assessment of defect severity. The methods tested included SVR, DTR, RFR, GBR, and FNN.

SVR was configured with a high regularization parameter (C = 100) to minimize errors, a gamma of 0.1 to balance the influence of training samples, and an epsilon of 0.1 to define the error margin. DTR operated without explicit depth limitations, allowing deep tree growth with a minimum of two samples required for a node split and one for leaf creation, enabling detailed pattern learning. RFR utilized 100 trees and automatically considered all features, enhancing robustness and prediction stability. GBR incorporated 100 sequential trees with a learning rate of 0.1 and a maximum tree depth of 3. Lastly, the FNN utilized a shallow architecture suitable for capturing the nonlinear relationships in the data without overfitting. Training involved monitoring the training and validation losses closely, with an initial learning rate of 0.003 optimized via the Adam optimizer across 40 epochs and a batch size of 16. To ensure robustness and prevent overfitting, 5-fold cross-validation was employed during training. The performance was tracked using validation accuracy at each epoch, serving as a criterion for early stopping to enhance model generalization for effectively quantifying the continuous assessment of defect depths in high-dimensional data transformed via PCA. Figure 14 shows the performance of the regression models for predicting the depth points onto the PCA space.

Regression analysis was performed using RMSE, R² and EVS as the metrics model accuracy and the proportion of variance in predicted depth. It is important to note that all performance metrics, including RMSE, R², and EVS, were calculated on the test set to ensure the results reflect the model’s ability to generalize to unseen data rather than being biased by the training set. The analysis in Table 4 revealed that while all models performed commendably, the Feedforward Neural Network and Decision Tree Regressor were particularly effective in predicting defect depths from spectral data. The NN recorded the best R² of 0.9757, suggesting that it could explain about 97.57% of the variance in the data, coupled with the lowest RMSE of 0.2829. The difference between R² and EVS could be due to the model being slightly overconfident in capturing the variability of the depth prediction.In summary, the FNN emerged as the most effective model for predicting defect depths from spectral data, with its combination of high accuracy, low error, and the ability to explain the largest proportion of variance. This showcases the potential of deep learning approaches in complex regression tasks. Through the regression analysis, the discrete categorization of defect depths (1–5 mm) was successfully extended to a continuous spectrum of depths. This transition from discrete to continuous modeling allows for a more nuanced and precise evaluation of defect severity.

**Table 4:** Results of the machine learning regression.

Method	RMSE	R$\phantom{0}^{2}$	EVS
Support vector regression	0.4054	0.9488	0.9488
Decision tree regression	0.2834	0.9750	0.9750
Random forest regression	0.2855	0.9746	0.9746
Gradient boosting regression	0.2848	0.9747	0.9747
Feedforward neural network	0.2829	0.9757	0.9768

Significant values are in underline.

## Conclusions

In this study, a Multi-Spectral Imaging setup was developed to observe a steel test specimen with artificial defects of differing depths and diameters at differing temperatures following a cooling excitation. The developed imaging system comprised a multi-spectral lighting system, a multi-spectral camera and an enclosure to house all components. The captured spectral data were subjected to pre-processing steps to improve the data’s SNR and reduce the effect of non-uniform spatial illumination caused by the multi-spectral lighting system. Spectrum signatures of the defected areas were generated, and PCA was applied to reduce data redundancy. Finally, a machine learning regression analysis was conducted to thus paving way to the metal loss defect quantification.

Spectrum signatures generated at all observed temperatures of 0–24 $\phantom{0}^{\circ }$C showed distinct bands for each defect depth. Among the observed wavelengths, the 700–900 nm was identified as the crucial wavelength span to give the best imaging data and clear separability of spectral bands of different defect depths. Furthermore, 735 nm was observed to have the highest clarity in images between defected and non-defected areas. Following the PCA dimension reduction on spectrum signature data, the trained machine learning models could identify the depth of metal loss defects to a satisfactory overall RMSE of 0.2829 with an R² of 0.9757, suggesting that it was able to explain approximately 98% of the variance in the data. Therefore, the accuracy is good enough for most on-field applications. It is important to note that, although the visible and UV spectra provided fewer distinct features (hue and contrast) for this sample, the results underscore the flexibility of the muti-spectral imaging approach. For other samples with different material properties or defect characteristics, these spectral ranges could play a significant role. The results highlight the potential advantages of MSI other imaging methods such as IRT in capturing comprehensive spectral information for a variety of materials and defect types.

## Data Availability

The datasets used and/or analysed during the current study available from the corresponding author on reasonable request.
